# Which is the Best Way to Perform the Physiological Cost Index in Active Individuals With Unilateral Trans-Tibial Amputation?

**DOI:** 10.33137/cpoj.v2i1.32953

**Published:** 2019-12-14

**Authors:** S Brunelli, A Sancesario, M Iosa, A.S. Delussu, N Gentileschi, C Bonanni, C Foti, M Traballesi

**Affiliations:** 1 Fondazione Santa Lucia, Scientific Institute for Research, Hospitalization and Health Care, Rome, Italy.; 2 Physical and Rehabilitation Medicine, Tor Vergata University of Rome, Rome, Italy.

**Keywords:** Oxygen Consumption, Prosthesis, Gait, Treadmill, Lower Limb Amputation, Physiological Cost Index, Energy Cost of Walking, Walking Test

## Abstract

**BACKGROUND:**

Physiological Cost Index (PCI) is a simple method used to estimate energy expenditure during walking. It is based on a ratio between heart rate and self-selected walking speed. Previous studies reported that PCI is reliable in individuals with lower limb amputation but only if there is an important walking impairment. No previous studies have investigated the correlation of PCI with the Energy Cost Walking (ECW) in active individuals with traumatic unilateral trans-tibial amputation, considering that this particular category of amputees has an ECW quite similar to healthy individual without lower limb amputation. Moreover, it is important to determine if PCI is also correlated to ECW in the treadmill test so as to have an alternative to over-ground test.

**OBJECTIVES:**

The aim of this study was to evaluate the correlation between PCI and ECW in active individuals with traumatic trans-tibial amputation in different walking conditions. The secondary aim was to evaluate if this correlation permits to determine ECW from PCI values.

**METHODOLOGY:**

Ninety traumatic amputees were enrolled. Metabolic data, heart rate and walking speed for the calculation of ECW and for PCI were computed over-ground and on a treadmill with 0% and 12% slopes during a 6-minute walking test.

**FINDINGS:**

There is a significant correlation between ECW and PCI walking over-ground (p=0.003; R^2^=0.10) and on treadmill with 12% slopes (p=0.001; R^2^=0.11) but there is only a poor to moderate correlation around the trendline. No significant correlation was found walking on treadmill with 0% slope. The Bland-Altman plot analysis suggests that is not possible to evaluate ECW directly from PCI.

**CONCLUSIONS:**

PCI is a reliable alternative measure of energy expenditure during walking in active individuals with trans-tibial amputation when performing over-ground or at high intensity effort on treadmill. PCI is therefore useful only for monitoring a within subject assessment.

## INTRODUCTION

Lower-limb amputees represent a particular group of interest in terms of gait, like previous studies have highlighted, as these persons use more energy compared to the healthy ones.^1-4^ Moreover, the energy cost and the effort required has been shown to be related to the level and the cause of the amputation: the higher the amputation level the greater the walking energy cost;^1,5,6^ further vascular disease amputees have higher walking energy expenditure than traumatic amputees.^7,8^

Oxygen consumption measurement (VO2) with a portable metabolimeter is the primary choice for assessing energy cost of walking (ECW) in amputees and it has been widely used in literature.^5,9^ However, it is time-consuming, the instrumentation needed is expensive and the methodology requires trained personnel. VO2 is the amount of oxygen taken up and utilized by the body mass per minute (ml/kg/min). ECW is the oxygen cost of walking and is defined as oxygen consumption related to walking speed: VO2 (ml/kg/min)/walking speed (m/min).^10^

Physiological Cost Index (PCI) considers heart rate as an indicator of energy expenditure. MacGregor has studied how PCI reflects the heart function and therefore indirectly the O2 consumption.^11^ The PCI is a valuable tool, that provides a simple, quick and inexpensive method to evaluate the O2 consumption during exercise^12,13^ and it is based on the linear relationship between VO2 and heart rate at submaximal workloads.^11^

PCI has been used as an outcome measure in many pathologies ^14-17^ and in elderly persons.^18^ In literature, several authors have used the PCI as an outcome measure to evaluate the energy expenditure of walking in lower limb amputees.^1-4,19-22^ Hagberg et al. have reported the test-retest reproducibility of the PCI between lower limb amputees and healthy persons, however there is no evidence of a linear correlation between PCI and ECW when performing high intensity effort.^3^ Chin et al. observed a significant correlation between PCI and oxygen uptake in trans-femoral amputees, however, the study was conducted on a small group of 6 unilateral trans-femoral amputees.^23^

About the correlation between PCI and ECW, Graham et al. reported that PCI scores did not correlate with VO2, indicating that the PCI is not a valid measure of energy expenditure in healthy persons. Walking at a comfortable pace for healthy persons provides only a minimal stress on the cardiovascular system and very low energy expenditure very near to resting values.^24^ Besides MacGregor indicated that PCI requires a submaximal effort.^11^ Considering that there should be a positive correlation between PCI and ECW only in the condition with a significant walking impairment, we hypothesize that active individuals with trans-tibial amputation (TTA) without any stump problems or clinical comorbidities were quite similar to healthy persons and therefore PCI could not be effective in the standard testing condition, i.e. during over-ground walking. In effect, ECW values in active TTA have shown small differences compared to those of healthy persons.^25,26^

Finally, PCI has been always calculated when the participants were walking over-ground: the large track needed for testing is not practical in all clinical setting and the presence of too many turns may influence the walking speed and consequently the PCI. A treadmill evaluation of PCI could be easier, especially for laboratories with small spaces.

No previous studies have investigated the correlation between PCI and ECW on active adult traumatic TTA. The primary aim of this study was to evaluate the reliability of PCI, compared to ECW, in this particular category of individuals during over-ground walking test (OWT), treadmill walking test with 0%. slope (TWT0%), and treadmill walking test with 12% slopes (TWT12%). This last test was performed with the hypothesis that when increasing the cardiopulmonary requests, there would be a stronger correlation between the two measures. The secondary aim was to evaluate if it is possible to determine ECW from PCI values of OWT.

## METHODOLOGY

### Study design:

Cross-sectional study

### Setting:

Research laboratory of Amputees Section of Operative Unit 4, Fondazione Santa Lucia, Rehabilitation Hospital, Rome.

### Sample:

The study sample included TTA. They were randomly selected from those who met the inclusion criteria in our database.

### Inclusion criteria:

1) unilateral traumatic TTA, 2) age 20-65 years old, 3) mass <, 116 kg, 4) use of the prosthesis for at least 18 months and for a minimum of 4 hours per day, 5) a mobility level of K3 or more based on the K-Levels^27^ (i.e. amputees that have the ability for ambulation with variable cadence, typical of community ambulatory or active adults), 6) absence of pathological stump condition that may affect prosthesis use, 7) absence of mental disorder, 8) absence of heart disease (except for hypertension well controlled by drugs) or respiratory disease 9) no usage of drugs that interferes with heart rate (i.e. Beta-blockers, digoxin), 10) the rehabilitative program and the prosthetic training were completed, 11) absence of pain or mobility deficit on sound limb. All participants gave their informed consent and they received no payment.

All TTA were fitted with a modular prosthesis with total surface bearing socket, passive vacuum suspension system and a dynamic-response foot.

The TTA performed a 6-minute walking test (6MWT) in three different conditions: one over-ground and two on a treadmill. The OWT was carried out in a 61-meter hallway with straight course and regular surface, walking back and forth at comfortable self-selected walking speed (SSWS).^26^ On the treadmill (RUNRACE, Technogym, Italy) the TWT0% and the TWT12%, were conducted with the speed indicator covered; each participant chose his SSWS without knowing the speed indicated on the treadmill.^26^ The walking tests were performed in the morning in three different days in a random sequence.

During all 6MWTs cardiac and metabolic data were collected with a portable gas analyzer K4b2 (Cosmed, Italy), that allowed a breath by breath recording of VO2 and heart rate. The time length of the 6MWT was enough for the TTA to reach the steady state phase of cardiac and metabolic data. Before each tests the TTA sat quietly for 10 minutes in order to collect rest heart rate data, for calculation of PCI. The data obtained at self-selected walking speed were used to calculate ECW and PCI.

The mean walking speed of the OWT was calculated as the ratio of distance to time in the steady state phase only. For the measurement of PCI and ECW, some precautions were taken: before the tests, enrolled TTA had to abstain from exhausting efforts, fatty foods, smoking and alcohol (the day before) and in the previous 60 minutes they were denied to take any kind of stimulants (tea, coffee, chocolate) or smoking.

The PCI and ECW data of each patient during the three walking conditions were calculated. The ECW was calculated using the formula “oxygen consumption/speed”. The formula used for PCI calculation was “walking heart rate – resting heart rate /speed”.

### Statistical Analysis

Pearson’s correlation coefficient (R) was computed to assess the association between the values of ECW and PCI data of each condition. The correlations between ECW and PCI between trials and participants within the same condition were computed. The possible agreement between PCI and VO2 was analyzed by means of Bland-Altman plot. The alpha-level of statistical significance was set at 0.05 for all the analysis. SPSS 17.0 software (SPSS, Inc., Chicago, IL) was used for all the statistical analysis.

## RESULTS

We evaluated 90 male participants whose characteristics are summarized in Table 1. None of the participants interrupted the test sessions due to fatigue.

**Table 1:** Demographic characteristics of the sample.

90 male trans-tibial amputees	Age (y)	Weight (kg)	Height (m)	Time since amputation (months)	K- LEVEL
**Mean**	46.3	86.3	1.7	114.5	3.6
**SD**	9.7	13.5	0.7	86.5	0.5

**Figure 1:**
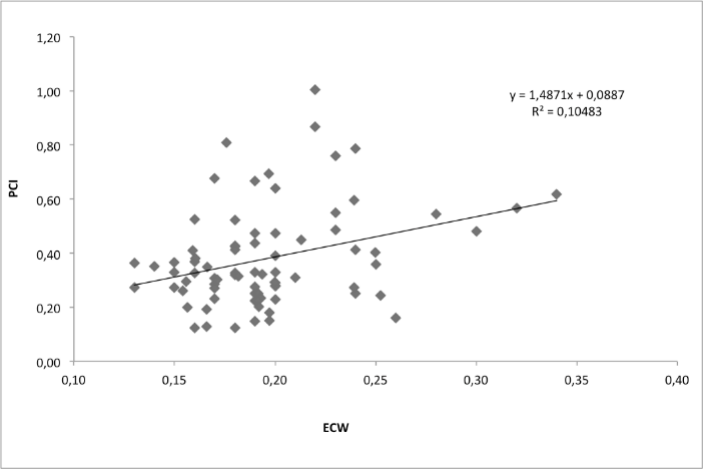
Correlation between PCI and ECW during over-ground walking test, (p=0.003).

The mean values of OWT were: ECW=0.195±0.039 ml/kg/m and PCI=0.380±0.183 (beats/m), p=0.003. (Figure 1). The chart shows that there is a positive correlation between the two parameters. The large inter-participant variation means that there is only a poor to moderate correlation around the trendline.

**Figure 2:**
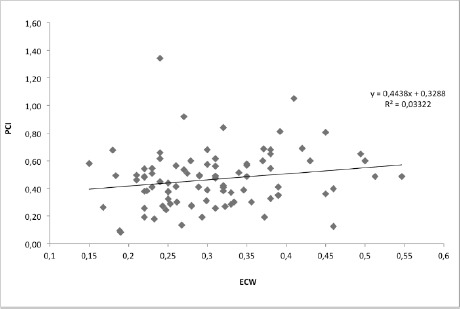
Correlation between PCI and ECW during treadmill walking test with 0% slope, (p=0.075).

The mean values of TWT0% were ECW=0.307±0.084 ml/kg/m and PCI=0.465±0.205 (beats/m), p=0.075 (Figure 2).

**Figure 3:**
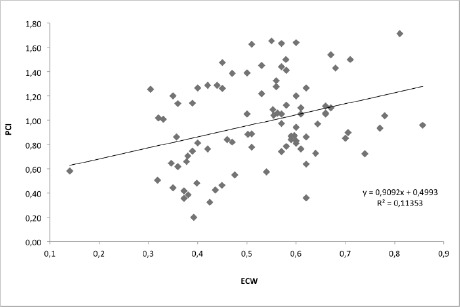
Correlation between PCI and ECW during treadmill walking test with 12% slope, (p=0.001)..

Last, in the third condition (TWT+12%) mean ECW was 0.525±0.132ml/kg/m, mean PCI was 0.977±0.355 (beats/m), p=0.001 (Figure 3). The chart shows that there is a positive correlation between the two parameters but there is only a poor to moderate correlation around the trendline.

**Figure 4:**
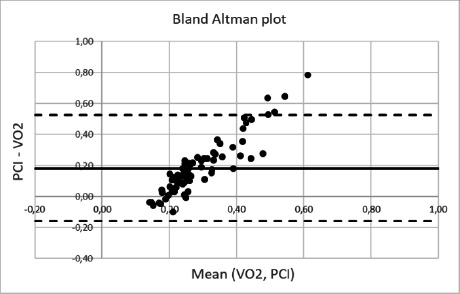
Bland-Altman plot of PCI and VO2 for over-ground walking.

Figure 4 shows, by means of the Bland-Altman plot, how the differences between PCI and VO2, evaluated for over-ground walking, vary with respect to their mean value.

## DISCUSSION

PCI is easy to calculate, is low-cost and no high technology equipment is required, but in order to start using it in trials as an alternative measure of ECW, it’s important to know their correlation and limits. This is the first study that compared ECW and PCI in a large sample of individuals with lower limb amputation.

The primary aim of this study was to evaluate the correlation between PCI and ECW in active traumatic TTA. Our results showed that there is not a statistically significant correlation between ECW and PCI during the TWT0% (p=0.075) while there is a significant correlation between ECW and PCI during the OWT (p=0.003). This may be explained by the Traballesi et al. findings where the authors reported that the over-ground test is the one that better reflects real walking with prostheses.^5^ Moreover, the same study demonstrated that amputees tested on treadmill have shown a significantly slower SSWS. The absence of correlation between PCI and ECW on treadmill with a slope of 0% might depend on a decrease of physiological expenditure as participants are walking slower. When the treadmill test was performed with higher intensity effort (TWT+12%), the correlation between ECW and PCI is statistically significant (p=0.001). The effort of walking with 12% inclination causes an increase of the heart rate. We can speculate that, on treadmill, the more intense the exercise, the higher the correlation between ECW and PCI.

Previous studies which examined the correlation between PCI and VO2 in healthy persons without gait impairment did not report any correlations.^3,24^ Compared with those studies, that investigated the PCI in healthy persons, we obtain a mean over-ground PCI value of 0.38, which is close but higher to Hagberg (PCI mean value=0.33) and Graham (PCI mean value=0.32). These PCI values are much lower in patients with great walking impairment such as spinal cord injury patients walking using a gait orthosis (PCI=1.97),^28^ or patients with stabilized hemiparesis (PCI=0.76),^29^ or trans-femoral amputees (PCI=0.55).^2^

PCI reproducibility in healthy individuals and lower limb amputees has been reported, but there is lack of evidence about its correlation with ECW.^2^ PCI is directly proportional to heart rate and inversely proportional to speed, so any situation influencing heart rate or speed could influence PCI. Healthy persons have a lower increase of working heart rate than persons with gait impairments because the cardiovascular stress is much lower.^24^ So studies that investigated PCI on healthy participants could not be indicative for participants with gait impairment. In our study we assessed only TTA whose amputation was due to traumatic injuries. This is because dysvascular amputees usually have comorbidities that could influence the general health of the patients and their heart rate. The TTA of our study have yet gait difficulties with respect to the healthy population, but they do not have other comorbidities. In this way we could evaluate the correlation between PCI and ECW having only the gait impairment as a difference between individuals with trans-tibial amputation and healthy participants. We can confirm our hypothesis that TTA without any other comorbidities are quite similar to healthy persons, so it is important to change the PCI treadmill testing condition towards a submaximal exercise to reach a significant correlation of PCI with ECW for clinical or evaluation purpose.

Ultimately the Bland-Altman plot shows two biases that do not allow to evaluate ECW directly from PCI. Firstly, the fact that the mean value of the difference between PCI and VO2 is different from zero revealed the presence of an absolute systematic difference between the two parameters. Then, the linear relationship clearly shows that the increment of this difference is proportional to the mean. These two biases support the idea of a relationship, but not an agreement, between PCI and ECW.

### Study limitations

In our study we have analyzed only traumatic transtibial amputees so these results should not be generalized to larger populations with amputation or to other persons with gait impairments due to other reasons. The participants were allowed to walk at different SSWS over the three different conditions which will invariably affect the energy cost of walking and the correlations between the parameters. The amputees, in fact, walk slower on the treadmill to reduce their metabolic energy expenditure. We did not select the over-ground SSWT for the treadmill speed because we have followed the methods of Traballesi’s paper in which the amputees chose their SSWS without knowing the speed indicated on the treadmill.^26^ Further studies should test PCI and ECW with other treadmill inclination in order to find the equation, if it exists, that permits to calculate the ECW from PCI data. Moreover, further studies are needed to confirm the suggestion that PCI has a higher correlation with the ECW when the walking effort increase.

## CONCLUSION

Our data allow us to state that PCI value do not permit to calculate ECW. Anyway the PCI can be used as an alternative measure of energy expenditure in active individuals with trans-tibial amputation for a within participant assessment only when recorded during over-ground walking or on treadmill with 12% slope. In reverse, the PCI does not correlate with ECW when recorded during treadmill walking with 0% slope. These findings have to be considered when PCI is used as a tool for estimate energy expenditure of walking.

## DECLARATION OF CONFLICTING INTERESTS

The authors have no conflicts of interest to declare.

## SOURCES OF SUPPORT

No funding for this study was provided.

## ETHICAL APPROVAL

The study approval was obtained by local ethics committee.

## AUTHOR CONTRIBUTION

- **Stefano Brunelli**,
  conceived the idea of the work, supported the data analysis and led the writing of the manuscript.
- **Andrea Sancesareo**,
  drafted the manuscript, managed the data files, interpretation of the data.
- **Marco Iosa**,
  conducted the statistical analyses, interpretation of the data.
- **Anna Sofia Delussu**,
  acquisition, analysis of the data.
- **Noemi Gentileschi**,
  supported the writing of the manuscript.
- **Cinzia Bonanni**,
  supported the writing of the manuscript.
- **Calogero Foti**,
  revised the manuscript critically for important intellectual content.
- **Marco Traballesi**,
  revised the manuscript critically for important intellectual content, design of the work.
